# Extracellular Vesicles Secreted from Cancer Cell Lines Stimulate Secretion of MMP-9, IL-6, TGF-β1 and EMMPRIN

**DOI:** 10.1371/journal.pone.0071225

**Published:** 2013-08-01

**Authors:** Jasmina S. Redzic, Agnieszka A. Kendrick, Karim Bahmed, Kristin D. Dahl, Chad G. Pearson, William A. Robinson, Steven E. Robinson, Michael W. Graner, Elan Z. Eisenmesser

**Affiliations:** 1 Department of Biochemistry and Molecular Genetics, University of Colorado Denver, School of Medicine, Aurora, Colorado, United States of America; 2 Department of Cell and Developmental Biology, University of Colorado Denver, School of Medicine, Aurora, Colorado, United States of America; 3 Division of Medical Oncology, University of Colorado Denver, School of Medicine, Aurora, Colorado, United States of America; 4 Department of Neurosurgery, University of Colorado Denver, School of Medicine, Aurora, Colorado, United States of America; University of Nebraska Medical Center, United States of America

## Abstract

Extracellular vesicles (EVs) are key contributors to cancer where they play an integral role in cell-cell communication and transfer pro-oncogenic molecules to recipient cells thereby conferring a cancerous phenotype. Here, we purified EVs using straightforward biochemical approaches from multiple cancer cell lines and subsequently characterized these EVs via multiple biochemical and biophysical methods. In addition, we used fluorescence microscopy to directly show internalization of EVs into the recipient cells within a few minutes upon addition of EVs to recipient cells. We confirmed that the transmembrane protein EMMPRIN, postulated to be a marker of EVs, was indeed secreted from all cell lines studied here. We evaluated the response to EV stimulation in several different types of recipient cells lines and measured the ability of these purified EVs to induce secretion of several factors highly upregulated in human cancers. Our data indicate that purified EVs preferentially stimulate secretion of several proteins implicated in driving cancer in monocytic cells but only harbor limited activity in epithelial cells. Specifically, we show that EVs are potent stimulators of MMP-9, IL-6, TGF-β1 and induce the secretion of extracellular EMMPRIN, which all play a role in driving immune evasion, invasion and inflammation in the tumor microenvironment. Thus, by using a comprehensive approach that includes biochemical, biological, and spectroscopic methods, we have begun to elucidate the stimulatory roles.

## Introduction

Cellular shedding is a process that occurs in all cells as a means to eliminate unneeded cellular components, yet the critical role of secreted vesicles in cell-cell communication is beginning to emerge [1], [2]. Membrane proteins are shed via a number of different mechanisms that include ectodomain shedding and secretion of full length membrane proteins via secreted vesicles [3]. Vesicular shedding occurs by outward budding of the plasma membrane with the release of a type of vesicle known as a microvesicle or by inward budding of the membrane with the eventual release of vesicles known as exosomes [4]. Here, we will collectively refer to both microvesicles and exosomes as extracellular vesicles (EVs). This phenomenon of vesicular shedding, i.e., EV shedding, has also been observed in a number of different diseases including neurological disorders, viral infection and cancer [5]–[7]. In fact, EV shedding occurs to a greater extent in cancer cells compared to healthy cells and results in the release of pro-oncogenic molecules including proteins, RNA and DNA [8], [9]. Recently, several roles of EVs have emerged that allow these particles to drive processes necessary for cancer development and progression such as angiogenesis, inflammation and drug resistance [10]. There are several mechanisms by which EVs may act on recipient cells. For example, these may include either direct stimulation of cellular receptors by proteins on the EV surface or internalization of EVs by the recipient cell, which both lead to subsequent stimulation of signaling pathways [1], [11]. Cancer cells appear to use EVs as a means of cell-cell communication by transferring their contents (DNA, RNA and protein) to a recipient cell, thereby leading to a transformation from a non-malignant to a malignant phenotype of the recipient cell [12]–[14]. The protein content of the EVs plays an integral part in the internalization and activity of the EVs. For example, EV proteins engage the recipient cells resulting in the uptake of EVs [15], and EVs were shown to transfer onco-proteins resulting in phenotypic change of the recipient cell [12]–[14]. However, one of the remaining questions with regard to EV activity is whether there are differences in the observed EV activity among the different recipient cell types as well as which proteins may be secreted by EVs. To this end, we assessed the differences in EV stimulatory activity in several different recipient cell types by probing EV-stimulated secretion of several different factors that have been shown to play significant roles in human cancers.

We have purified EVs from healthy individuals, cancer patients and from several different mammalian cancer cell lines. Our purification method yielded a heterogeneous population of EVs ranging in size from 20–300 nm indicating a mixture of exosomes and microvesicles [4]. We detected the full length transmembrane protein called Extracellular Matrix MetalloPRoteinase Inducer (EMMPRIN), a proposed marker of EVs [16], in EVs purified from several different biological fluid samples and from all the different cells lines we evaluated here. We, therefore, used EMMPRIN as a marker for our purified EVs. Fluorescence microscopy showed that our purified EVs were internalized by the recipient cell in a relatively short time, (5–15 minutes), and localize around the nucleus, thereby confirming that our purification method resulted in active EVs capable of transferring their contents into recipient cells. Our broad based evaluation of several different recipient cell lines shows that EVs purified from these various types of cancer cells exhibit preferential stimulatory activity of secreted proteins toward monocytes but not epithelial cells. In fact, while we discovered that EVs secreted from all cell lines studied here contain full length EMMPRIN, EVs also stimulate the secretion of full length EMMPRIN itself in human monocytic leukemia cells. We discovered that EVs are potent stimulators of Transforming Growth Factor Beta-1 (TGF-β1), Matrix Metalloproteinase-9 (MMP-9) and Interleukin-6 (IL-6). This stimulatory activity of EVs to induce secretion of TGF-β1, MMP-9, IL-6 and EMMPRIN, suggests a role of the EVs in driving tumor progression by stimulating factors important for immune evasion, invasion and inflammation [17]–[19]. Our data brings us closer to a better understanding of the biology of secreted EVs, the preferential cell type being stimulated by EVs, and the molecules that are secreted by recipient cells upon stimulation with secreted EVs.

## Materials and Methods

### Ethics Statement for Human (patient/donor) Collection of Biological Fluids

Peripheral blood samples were collected in the University of Colorado Hospital Cutaneous Oncology Clinic. Vacutainers tubes (red top, Becton Dickinson, Franklin Lakes, NJ, USA) were used for serum collection. Samples were immediately transported to lab, centrifuged at 1200×g for 10 minutes then frozen at –80°C until needed. Lithium heparin containing vacutainers (green top, Becton Dickinson, Franklin Lakes, NJ, USA) were used for plasma collection, immediately transported to the lab, centrifuged at 800×g for 10 minutes at 4°C. Plasma samples were frozen at –80°C until needed. Ascites fluid was collected from pleural effusion taps in Interventional Radiology at the University of Colorado Hospital using a vacutainer (red top, Becton Dickinson, Franklin Lakes, NJ, USA). Samples were immediately transported to the lab on ice, centrifuged at 800×g for 5 minutes at 4°C to removed cellular debris. Samples were frozen at −80°C until needed. The collection of biological fluid samples was approved by the Colorado Multi-Institutional Review Board (COMIRB # 05-0309), and samples were collected following informed written consent.

### Detection of Full Length EMMPRIN in Biological Fluids

Human serum, plasma and ascites fluid samples were filtered using a 0.22 µm filter to remove any cells and cellular debris. 10 µg of Human EMMPRIN Biotinylated polyclonal antibody (R & D Systems, Minneapolis, MN, USA) was used to immunoprecipitate (IP) EMMPRIN from each sample. The IP was carried out for 2 hours at 4°C with rotation followed by incubation with 40 µl of streptavidin beads (GE Healthcare, Piscataway, NJ, USA) for 2 hours at 4°C with rotation. PNGaseF (New England Biolabs, Ipswich, MA, USA) was used to deglycosylate the IPed fraction in a 1∶10 enzyme to sample ratio as per the manufacturer’s protocol. 5 µl of 10 X SDS Loading Buffer was added to the sample and boiled for 10 minutes prior to loading on a 4–12% Bis-Tris SDS-PAGE gel (Invitrogen, Grand Island, NY, USA) then transferred to an Immobilon Polyvinylidene fluoride (PVDF) membrane (Millipore, Bedford, MA, USA) for Western blot analysis. The membrane was blocked in 2% milk for 30 minutes then incubated in human EMMPRIN monoclonal antibody (R & D Systems, Minneapolis, MN, USA) prepared using a 1∶1000 dilution in 2% milk for 1 hour. Following the primary antibody incubation, the membrane was washed with 1 X Phosphate Buffered Saline (PBS) for 30 minutes. Anti mouse IgG conjugated to HRP (Cell Signaling Technology, Danvers, MA, USA) was prepared using a 1∶4000 dilution, applied to the membrane and incubated for 1 hour followed by an additional wash in 1 X PBS for 30 minutes. Protein band detection was performed with the ECL chemiluminescence detection kit using a Perkin Elmer detection kit (Perkin Elmer, Waltham, MA, USA).

### Culturing of Cells

MCF-7 cells were a kind gift from Dr. Heide Ford (Department of Pharmacology, University of Colorado Denver School of Medicine) [20]. MDA-MB-231 cells were a kind gift from Dr. Jennifer Richer (Department of Pathology, University of Colorado Denver School of Medicine) [21]. U937, THP-1, Molm13 and Human Foreskin Fibroblast (HFF) cell lines were a kind gift from Dr. James DeGregori (Department of Biochemistry and Molecular Genetics, University of Colorado Denver School of Medicine) [22]–[24]. L3.6pL cells were a kind gift from Dr. Isaiah J. Fidler (Department of Cancer Biology, The University of Texas MD Anderson Cancer Center) [25]. All cells were cultured at 37°C with 5% CO_2_. MCF-7 and MDA-MB-231 cells were cultured in DMEM media supplemented with high glucose, L-glutamine, sodium pyruvate, 1% penicillin/streptomycin/amphotericin-b and 10% fetal bovine serum (FBS; Fisher Scientific, Pittsburgh, PA, USA). L3.6pL cells were cultured in DMEM media supplemented with L-glutamine, sodium pyruvate, 5 mM non-essential amino acids, 25 µg/mL plasmocin, 1% penicillin/streptomycin/amphotericin-b and 10% FBS. U937, Molm13, THP-1 and HFF cells were cultured in RPMI 1640 media supplemented with L-glutamine, 1% penicillin/streptomycin/amphotericin-b and 10% FBS.

### Purification of EVs

Adherent cell lines (MCF-7, MDA-MB-231, L3.6pL and Hek293Fpl) used for vesicle purification were cultured in 150 mm plates until the cells were 70% confluent. Media was removed and the cells were washed with 1 X PBS two times. Appropriate media supplemented with 3% FBS depleted of EVs via ultracentrifugation was added to the cells [26].

EVs from 5×10^5^ cells/mL of U937 cells were cultured in T-75 flasks in RPMI 1640 media and 3% vesicle free FBS. At collection time, cells were pelleted by centrifugation for 5 minutes at 1100 rpm and the supernatant was collected. The supernatant from all cells was collected every 48 hours and 4 mM Ethylenediaminetetraacetic acid (EDTA) (Fisher Scientific, Pittsburgh, PA, USA) was added to the supernatant immediately upon collection. The supernatant was centrifugated at 8,000 rpm for 10 minutes to remove any cellular debris and then filtered using a 0.22 µm filter. Using a 100 kDa molecular weight cut off filter membrane (Millipore, Bedford, MA, USA) the supernatant was concentrated to a 1–2 mL volume. EVs from the concentrated sample were purified using a preparatory Superose 6 size exclusion chromatography column (GE Healthcare, Piscataway, NJ, USA) equilibrated in 1 X PBS, pH 7.5. Total protein content of purified EVs was measured using the Bradford total protein assay (BioRad, Hercules, CA, USA).

### Identification of the Full Length EMMPRIN within Purified EVs

The void volume fractions from the size exclusion chromatography were pooled and concentrated to 500 µl. 10 µg of Human EMMPRIN Biotinylated polyclonal antibody (R & D Systems, Minneapolis, MN, USA) was used to immunoprecipitate (IP) EMMPRIN from each sample. The IP was carried out for 2 hours at 4°C with rotation followed by incubation with 40 µl of streptavidin beads (GE Healthcare, Piscataway, NJ, USA) for 2 hours at 4°C with rotation. PNGaseF (New England Biolabs, Ipswich, MA, USA) was used to deglycosylate the IPed fraction in a 1∶10 enzyme to sample ratio as per the manufacturer’s protocol. 5 µl of 10 X SDS Loading Buffer was added to the sample and boiled for 10 minutes prior to loading on a 4–12% Bis-Tris SDS-PAGE gel (Invitrogen, Grand Island, NY, USA) then transferred to an Immobilon Polyvinylidene fluoride (PVDF) membrane (Millipore, Bedford, MA, USA) for Western blot analysis. The membrane was blocked in 2% milk for 30 minutes then incubated in human EMMPRIN monoclonal antibody (R & D Systems, Minneapolis, MN, USA) prepared using a 1∶1000 dilution in 2% milk for 1 hour. Following the primary antibody incubation, the membrane was washed with 1 X Phosphate Buffered Saline (PBS) for 30 minutes. Anti mouse IgG conjugated to HRP (Cell Signaling Technology, Danvers, MA, USA) was prepared using a 1∶4000 dilution, applied to the membrane and incubated for 1 hour followed by an additional wash in 1 X PBS for 30 minutes. Protein band detection was performed with the ECL chemiluminescence detection kit using a Perkin Elmer detection kit (Perkin Elmer, Waltham, MA, USA).

### Mass Spectrometry Analysis of the Secreted EVs

Following the IP of EMMPRIN from the serum samples, 10 µl of sample was electrophoresed using a 4–12% Bis-Tris SDS-PAGE gel. Gel bands for mass spectrometry were visualized with Comassie blue dye. A band in the range of 25–35 kDa was excised from the gel and cut into 1 mm squares. The gel band was then rinsed several times in 25 mM Ammonium Bicarbonate/50% Acetonitrile (25 mM ABC/50% ACN) solution to remove excess Comassie blue stain. Sample was reduced with 1 mM Dithiothreitol (DTT, Gold Biotechnology, St. Louis, MO, USA) for 30 minutes at 70°C and then alkylated with 20 mM Iodoacetamide (IA, Sigma-Aldrich, St. Louis, MO, USA) for 45 minutes at room temperature in the dark. Samples are washed in double distilled water for 15 minutes while mixing (vortex), followed by 25 mM ABC/50% ACN wash and finally 100% ACN wash. Samples were then dried using a speed vacuum system then 10 µl of 10 mg/mL of Trypsin (Promega, Madison, WI, USA) was added to the gel bands and digestion was allowed to proceed over night at room temperature. Samples were analyzed on a LTQ-FT Ultra hybrid mass spectrometer (Thermo Fisher, Bremen, Germany). Peptide desalting and separation was achieved using a dual capillary/nano pump HPLC system (Agilent 1200, Palo Alto, CA, USA). The nano-pump run was 60 minutes at a flow rate of 350 nL/min. A 41 minute gradient from 12% ACN to 35% ACN was used to separate the peptides. The LC run was monitored by sequentially recording MS scans, in the ICR cell, while three MS2 scans were obtained in the ion trap via CID. Raw distiller (UCSF, CA, USA) was used to create de-isotoped, centroided peak lists from the raw spectra (.mgf format). These peak lists were searched against all human entries in the SwissProt protein database using Mascot™ server (Version 2.2, Matrix Science). For searches, mass tolerances were +/−10 ppm for MS peaks, and +/−0.6 Da for MS/MS fragment ions. Trypsin specificity was used allowing for 1 missed cleavage. The modifications of Methionine oxidation, protein N-terminal acetylation and peptide N-terminal pyroglutamic acid formation were allowed for. An expect value of <0.05 was considered significant.

### NanoParticle Tracking Analysis Measurement

NanoParticle tracking analysis (NTA) is one of the methods used for detecting secreted EVs within a given sample. Superose 6 size exclusion chromatography high molecular weight fractions were assayed using NTA Version 2.2 Build 0375 instrument (NanoSight, Amesbury, Wiltshire, United Kingdom). Samples were assayed at 1∶10,000 dilution and 1 mL of the diluted sample was used for analysis.

### Fluorescence Microscopy

1×10^6^ cells/mL cells were centrifuged at 1100 rpm for 5 minutes to pellet the cells. Cells were fixed with 200 µl of 1% Formalin (Fisher Scientific, Pittsburgh, PA, USA) for 10 minutes at room temperature. Cells were centrifugated as above, the supernatant removed and cells washed with 1 X PBS two times. Cells were incubated in 20 µl of EMMPRIN-FITC conjugated antibody (Ancell, Bayport, MN, USA) prepared at a 1∶50 dilution and incubated at room temperature for 15 minutes. Cells were washed with 1 X PBS twice following the incubation period. Nuclear staining was performed at room temperature for 10 minutes using 1 µl/mL of 4′,6-diamidino-2-phenylindole (DAPI) stain (Invitrogen, Grand Island, NY, USA). Cells were washed two times with 1 X PBS and mounted onto microscope slides.

To assess the interaction and localization of EVs with THP-1 cells, EVs were incubated with Texas Red stain for 30 minutes at room temperature in the dark. EVs were centrifugated for 90 minutes at 100,000 xg in the TLA-55 rotor. Supernatant was aspirated and the pellet washed in 1 X PBS then spun again as above 2 times. Supernatant was removed and the pellet resuspended in 20 µl of 1 X PBS. Stained EVs were then added to the cells and incubated at various times ranging from 5 minutes up to 24 hours at 37°C. The cells were visualized using a Nikon Ti Eclipse (Nikon Instruments Inc., Melville, NY, USA) inverted microscope with a Nikon 100X PlanApo NA 1.4 objective. Images were captured with an Andor iXon EMCCD 888E camera (Andor Technologies, South Windsor, CT, USA). Image analysis and quantification was performed using the NIS Elements imaging software (Nikon Instruments Inc., Melville, NY, USA). All images taken were acquired at room temperature. Acquisition times for images were 80 msec, 500 msec and 300 msec for the blue, green and red channels, respectively. Images were processed for figures using ImageJ and then produced using Corel Draw.

### Measuring the Change in EMMPRIN Expression Upon Vesicle Stimulation

Flow cytometry analysis of THP-1 cells stained with EMMPRIN-FITC conjugated antibody was performed to determine if there are any changes in the level of cell surface EMMPRIN expression upon treatment with EVs. EMMPRIN mean fluorescent intensity (MFI) was measured using the Beckman Coulter Cell Lab Quanta SC Flow Cytometer on untreated cells, cells treated with 1 X PBS (buffer treated cells) and EV treated cells. All measurements were taken 24 hours post stimulation and were performed using the entire cell population, i.e. 1×10^6^ cells/mL stimulated for 24 hours.

### Measuring the Change in EMMPRIN Secretion Upon Vesicle Stimulation

The supernatant from the control, buffer treated and EV treated samples was collected to determine if there are any changes in the amount of secreted EMMPRIN upon stimulation. 100 µl of the supernatant was deglycosylated as described earlier, and the proteins separated using SDS-PAGE electrophoresis. EMMPRIN secretion was measured using Western blot analysis as described earlier.

### Activity Assays

Secretion of MMP-9 and IL-6 was measured in multiple cell lines using Enzyme Linked Immunosorbent Assay (ELISA) detection kits (ELISA Tech, Aurora, CO, USA). For cells in suspension, 5×10^5^ cells/ml were used per 0.5 mL of media in a 12-well plate. For adherent cells, stimulations were carried out in 12-well plates with cells at 70–80% confluency. All activity assays were performed in serum free media. Supernatant from the stimulated cells was collected at 24 hours post stimulation and stored in the −20°C until needed. 100 µl of the supernatant was applied to the ELISA plate. TGF-β1 is secreted in the latent form, therefore, samples were acidified to pH 2 using 1 M Hydrochloric acid (Fisher Scientific, Pittsburgh, PA, USA) and incubated for an hour at room temperature to generate the immunoreactive form of TGF-β1. The samples were then neutralized with 1 M Sodium Hydroxide (Fisher Scientific, Pittsburgh, PA, USA). 100 µl of the activated sample was applied to the ELISA plate. Measurements were carried out as per the manufacturer’s protocol (ELISA Tech, Aurora, CO, USA).

## Results

### Full Length EMMPRIN Serves as a General Marker of EVs Secreted in Biological Fluids as Well as from Cultured Cells

The presence of extracellular EMMPRIN form(s) was first assessed in several types of biological fluids including human sera, plasma, and ascites fluid to determine whether EMMPRIN may be used as a general marker for the presence of EVs as has been recently suggested [16]. Samples were filtered using a 0.22 µm filter to remove any cells or cellular debris and immunoprecipitated (IPed) using an EMMPRIN antibody. Upon deglycosylation, the full length form of the protein (28 kDa) was detected in human sera and plasma from both healthy donors and cancer patients as well as in the ascites fluid collected from cancer patients with no observable levels of any cleaved forms within these biological fluids (see Fig. S1A and Fig. S1B for human sera and ascites fluid, respectively). Mass spectrometry analysis of tryptic digests was used to unambiguously confirm that EMMPRIN is in the IPed serum sample, Fig. S1C. Several peptides mapping to the ectodomain of EMMPRIN were detected and used to confirm the presence of extracellular EMMPRIN within the serum samples, Fig. S1C. The presence of a full length transmembrane protein such as EMMPRIN identified here is consistent with a general secretion of transmembrane proteins via EVs in all biological fluids, yet this was further shown in what follows.

In order to provide a consistent and reliable source of EVs for subsequent assays to directly probe their stimulatory roles, EVs were purified from several different mammalian cancer cell lines. EMMPRIN was probed to determine whether this transmembrane protein could be used as a general marker of EVs for all these cell lines analogous to EVs purified from biological fluids as above. Two breast cancer cell lines, MCF-7 and MDA-MB-231, were used and EVs purified from those cell lines are denoted as EV^MCF-7^ and EV^MDA^, respectively. In addition, both a monocytic leukemia cell line, U937, and a pancreatic cancer cell line, L3.6pL, were used with EVs derived from these cell lines denoted as EV^U937^ and EV^L3.6pL^, respectively. Supernatants from cells cultured in the appropriate media supplemented with antibiotics and 3% EV free FBS were collected every 48 hours.

**Figure 1:**
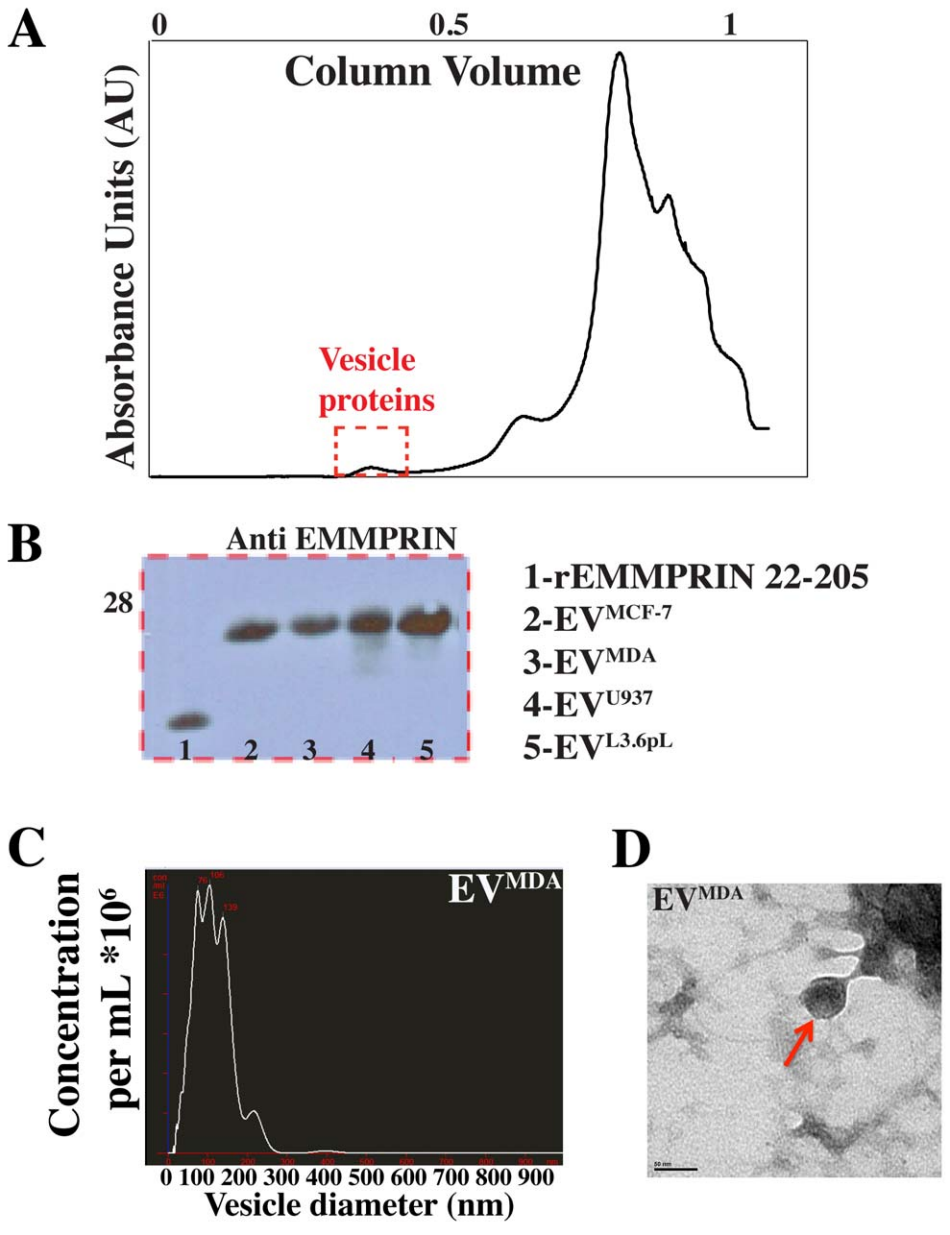
Purification of secreted EVs. A) Size exclusion chromatography elution profile of the purification of conditioned media to isolate EVs. The red box corresponds to the elution peak for the purified EVs. B) EMMPRIN is secreted via EVs in all cell lines tested as shown by the Western blot probed for EMMPRIN in the vesicle fractions purified using size exclusion chromatography. C) NanoParticle Tracking analysis detects EVs in the purified sample and validates our size exclusion method to purify EVs from conditioned media. D) Electron microscopy was used to visualize the secreted EVs. Data shown is for EV^MDA^.

Of the previously used methods employed to purify secreted EVs, we chose to use size exclusion chromatography [27]. Specifically, Superose 6 size exclusion chromatography was used to select for the large molecular weight fractions, Fig. 1A (red box). EMMPRIN was detected in the EV fraction collected from all of the cell lines tested here, Fig. 1B, indicating that EMMPRIN secretion is a widely occurring process.

Thus, our studies here indicate that extracellular, full length EMMPRIN secretion via EVs is a widely occurring phenomenon rather than a cell type specific process and EMMPRIN may be used as a general marker for the presence of EVs.

### Confirmation and Validation of Purified EVs using NanoParticle Tracking Analysis, Electron Microscopy and Mass Spectrometry

We used several methods commonly employed for EV detection and visualization to further validate our EV purification method beyond our confirmed presence of EMMPRIN as a general EV marker (Fig. 1A,B). Specifically, NanoParticle tracking analysis (NTA) was used for determination of the size distribution of EVs in samples. Based on the size distribution of the EVs detected using NTA (Fig. 1C), it can be concluded that the cell lines used in this study secrete a heterogeneous population of EVs ranging in size from 20–300 nm. The size range indicates that these vesicles comprise smaller EVs, known as exosomes (10–100 nm), and larger EVs known as microvesicles (100–1000 nm). Note that further sucrose gradient fractionation and subsequent Western blot analysis identified EMMPRIN in both EV populations (data not shown), indicating that EMMPRIN is a general marker for both types of EVs. Electron microscopy (EM) was used to visualize the EVs secreted from different cell lines as shown for purified EV^MDA^ (Fig. 1D). The EM data also showed heterogeneity in the vesicle size as was determined with NTA. Mass spectrometry analysis also yielded identification of several other EV markers such as CD63, CD81 and Annexin V as shown in Table 1, thereby further validating the purification method used here. Thus, given the size distribution detected with NTA and EM and the identification of EV specific proteins, we conclude that our purification method yields a mixture of exosomes and microvesicles.

**Table 1:** Mass Spectrometry identification of EV proteins.

Accession Number	Protein	Number of unique peptides	% sequence coverage
P08962	CD63	9	23
P60033	CD81	8	25
P21926	CD9	3	15
P08758	Annexin V	3	12
P62937	PPIA (Cyclophilin A)	6	51

* Protein identification was performed using 99% confidence threshold.

† Protein identification required at least two unique peptides.

### EVs are Rapidly Internalized into Recipient Cells

**Figure 2:**
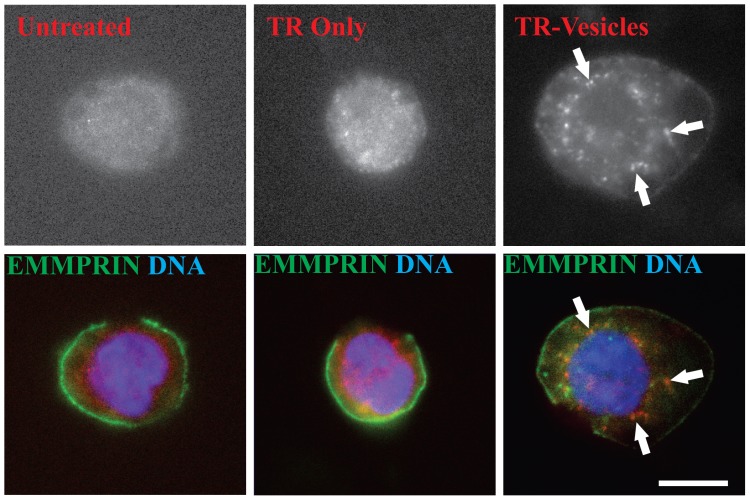
EVs are internalized into THP-1 cells. Fluorescence microscopy images of THP-1 cells treated with Texas red stained EVs. Left panel - THP-1 cell stained with EMMPRIN FITC and DAPI. The red signal is the auto-fluorescence signal of the cell. Middle panel - THP-1 cell treated with the Texas Red stain alone. The signal observed is the same as in the left image and corresponds to the Texas red background and cell auto-fluorescence signal. Right panel - THP-1 cells treated with Texas Red stained EVs and visualized after a 5-minute incubation period. The cell membrane is stained with EMMPRIN-FITC antibody, the EVs are shown in red and the DAPI stained nucleus is shown in blue. The reference bar is 10 µm.

EV modulation of recipient cell function has been proposed to be at least partially dependent on their initial uptake and transfer of cargo (see review by Lopez-Verrilli [28]). In accord with such a proposed mechanism, we found that the purified EVs are internalized within minutes by monocytic leukemia THP-1 cells (Fig. 2) and monocytic leukemia U937 cells (Fig. S4) thereby confirming the biological activity of our purified EVs. Specifically, we visualized the internalization of Texas Red stained EVs as distinct puncti into cells stained with an EMMPRIN-FITC antibody that allowed for visualization of the cellular membrane (Fig. 2, right). Thus, in these experiments we utilized the fact that EMMPRIN is initially highly expressed on the recipient cell surface as a marker for the cellular membrane. Single images from through cell volume Z-stacks were collected to confirm that the EVs are indeed internalized rather than simply localized on the cell surface. We also quantified the percentage of cells that internalized the EVs by counting the frequency of EV positive cells. Data averaged from three independent measurements shows that vesicle internalization occurs in 76% of the cells. Thus, vesicle internalization is not a rare occurrence but a frequent event. These data also show localization of Texas Red stained EVs on the cellular membrane, which indicates that EVs are likely incorporated into or associate with the cellular membrane as well as being internalized.

### EVs Stimulate Secretion of Several Cancer-associated Factors

#### Initial activity assays

We initially screened several recipient cell lines to determine which, if any, cultured cells were responsive to our purified EV^MCF^ and EV^MDA^. Specifically, MMP-9 and IL-6 secretion (Fig. S2 and Fig. S3, respectively) was monitored upon stimulation with EV^MCF-7^ and EV^MDA^ using Enzyme Linked Immunosorbent Assay (ELISA), since both of these proteins are upregulated in multiple cancers along with the EMMPRIN marker of EVs [29], [30]. In general, our data shows that there is preferential selectivity in the cell type eliciting a response upon EV stimulation. For example, the U937 monocytic cells were more responsive in secreting both IL-6 and MMP-9 upon stimulation with EVs when compared to epithelial cells. We did observe an increase in IL-6 secretion in both HFF and MBA-MB-231 cells upon stimulation with EV^MDA^, however, neither secretion of IL-6 or MMP-9 was stimulated with EV^MCF-7^. Thus, since U937 cells elicited the most notable response upon stimulation with both types of EVs used in these initial ELISA assays, we focused on monocytic cells, THP-1 and U937, in subsequent activity assays.

#### Purified EVs stimulate the secretion of EMMPRIN suggestive of a positive feedback loop

**Figure 3:**
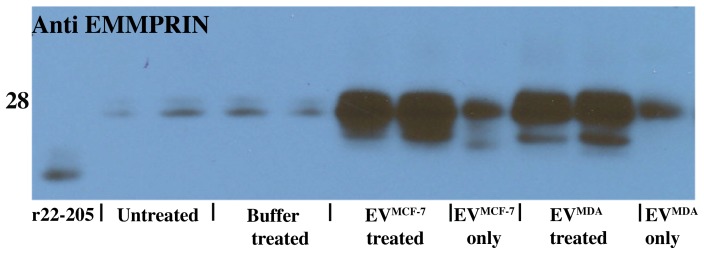
EMMPRIN is secreted upon stimulation with EVs. Western blot analysis of THP-1 cells shows the change in EMMPRIN secretion with and without stimulation with EV^MCF-7^ and EV^MDA^.

Since previous studies have shown that EMMPRIN is involved in a positive feedback loop in some cell lines [31], we next wanted to assess the effect of the EVs on EMMPRIN cell surface expression and secretion in monocytes. THP-1 cells were stimulated with 10 µg of total protein for each type of vesicle, i.e. EV^MCF-7^ and EV^MDA^, and incubated at 37°C for 24 hours. We detected a clear increase in full length EMMPRIN **secretion** upon stimulation with EVs when compared to EVs only, untreated cells, and buffer treated cells (Fig. 3). In contrast to vesicle-induced secretion of EMMPRIN, the level of **cell surface** EMMPRIN remained similar as measured by flow cytometry (data not shown). Interestingly, some lower molecular weight bands were also observed in some of the stimulated samples indicating that a small portion of total EMMPRIN is cleaved (see Fig. 3, EVs alone versus cells treated with EVs). Since the cleaved forms migrate significantly higher than the recombinant ectodomain (Fig. 3, lane 1) and the antibody recognizes the N-terminal Ig1 domain of EMMPRIN, these smaller forms are likely C-terminal truncations. Thus, EV^MCF-7^ and EV^MDA^ stimulate the secretion of EMMPRIN, and to a larger degree, full length EMMPRIN that we have shown is found in EVs, suggestive of a positive feedback loop for EV formation.

#### Cancer associated proteins are upregulated upon EV stimulation

We proceeded to assess the role of these purified EVs on monocytic cell lines. Specifically, we focused on MMP-9 and IL-6, since these two proteins are stimulated by several proteins found in EVs that include the EMMPRIN EV marker [32], [33]. We also monitored the change in TGF-β1 secretion given that this protein has recently been shown to play a role in modulating monocytic cell differentiation towards a more tumorigenic phenotype [17]. For example, TGF-β1 regulates the phenotypic change of monocytes to myeloid derived suppressor cells, which were shown to inhibit the immune response [34]. Secretion of MMP-9, IL-6 and TGF-β1 was measured upon stimulation with EVs using ELISA assays. However, we found that background levels of TGF-β1 were significantly elevated in U937 cells. Therefore, we turned our focus to THP-1 monocytic cells that exhibited low background levels of MMP-9, IL-6, and TGF-β1. We also extended our studies from EVs purified from breast cancer cells to EVs purified from other cell lines as well. In general, the purified EVs were active in eliciting secretion of all three of the proteins we evaluated from THP-1 cells and are discussed below in the context of each stimulated protein. Stimulations were performed in two independent experiments and similar results were observed.

**Figure 4:**
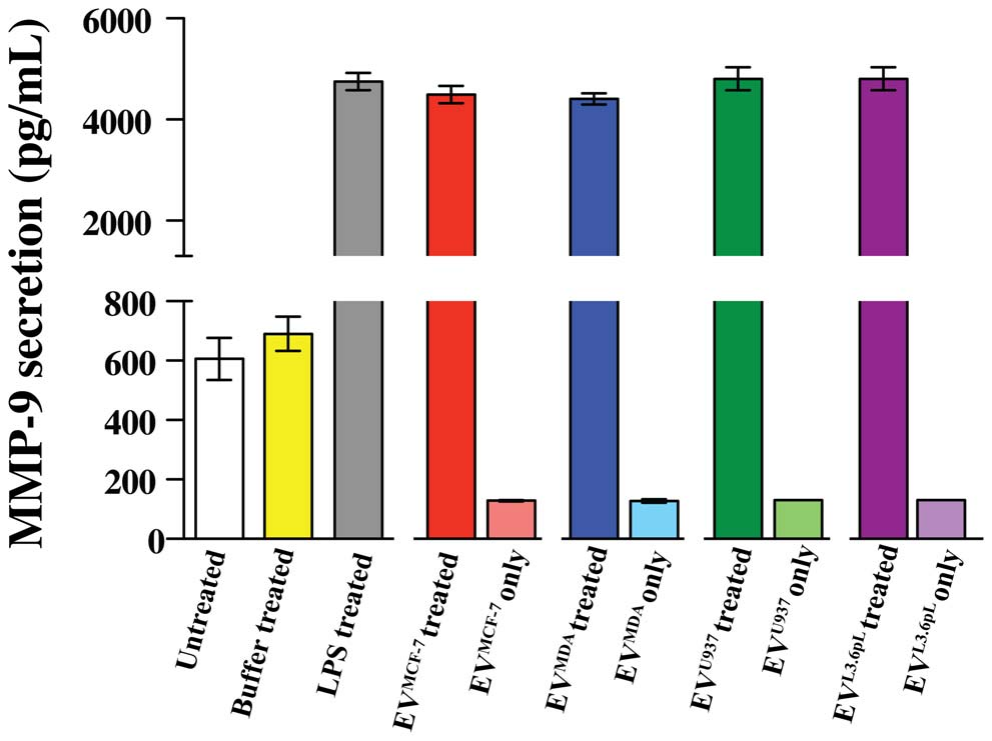
MMP-9 is secreted upon stimulation with EVs in THP-1 cells. MMP-9 secretion in THP-1 cells was measured with an ELISA assay upon stimulation with EV^MCF-7^, EV^MDA^, EV^U937^ and EV^L.6pL^. The EVs themselves secrete low levels of MMP-9 that did not contribute significantly to the overall observed secreted MMP-9 levels.

A robust secretion of MMP-9 from THP-1 cells was observed upon stimulation with all EVs when compared to buffer treated cells (Fig. 4). The EVs themselves had minimal background levels of MMP-9 and, thus, we can conclude that these EVs are potent stimulators of MMP-9 secretion from THP-1 cells.

**Figure 5:**
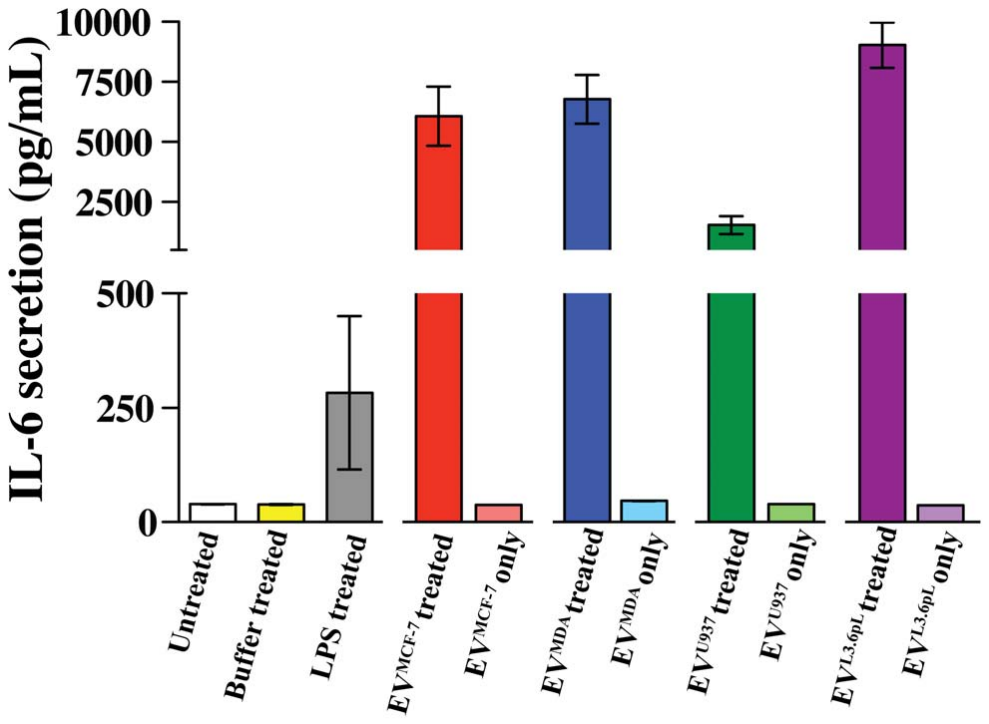
IL-6 is secreted upon stimulation with EVs in THP-1 cells. IL-6 secretion in THP-1 cells was measured with an ELISA assay upon stimulation with EV^MCF-7^, EV^MDA^, EV^U937^ and EV^L.6pL^. The EVs secrete low levels of IL-6 that did not contribute significantly to the overall observed secreted IL-6 levels.

We next measured the level of IL-6 secretion in THP-1 cells. As shown in Fig. 5, THP-1 cells secrete high levels of IL-6 upon stimulation with EVs, however, some variability was observed in response to different EVs. For example, stimulation of THP-1 cells by EV^MCF-7^, EV^MDA^ and EV^L3.6pL^ resulted in high levels of IL-6 secretion (Fig. 5, red, blue and dark purple bar, respectively). EV^U937^ also stimulated secretion of IL-6, but to a lesser extent than the other EVs used in this experiment (Fig. 5, dark green bar). Again, the background levels of IL-6 measured using the EVs alone were minimal and did not contribute to the observed secreted levels allowing us to conclude that EVs are potent stimulators of IL-6 secretion from THP-1 cells along with MMP-9.

**Figure 6:**
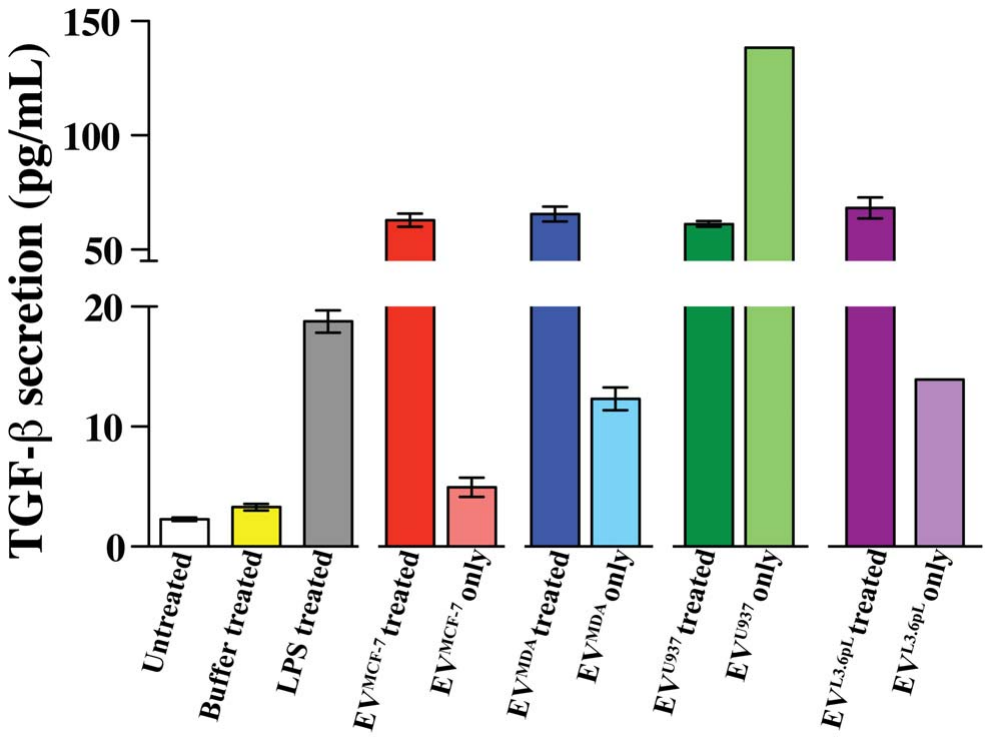
TGF-β1 is secreted upon stimulation with EVs in THP-1 cells. TGF-β1 secretion in THP-1 cells was measured with an ELISA assay upon stimulation with EV^MCF-7^, EV^MDA^, EV^U937^ and EV^L.6pL^. Most purified EVs secrete low levels of TGF-β1 that did not contribute significantly to the overall observed secreted TGF-β1 levels. Interestingly, EV^U937^ alone comprised high levels of TGF-β1 that were reduced upon incubation with THP-1 cells, potentially suggesting internalization of TGF-β1 that is well known [36].

Next, we measured the level of TGF-β1 secretion in THP-1 cells upon stimulation with EVs. THP-1 cells stimulated with EV^MCF-7^ and EV^MDA^ (Fig. 6, red and blue bar, respectively) showed a significant increase in TGF-β1 when compared to untreated and buffer treated cells. Interestingly, low levels of TGF-β1 were measured on the EVs themselves (Fig. 6, pink and aquamarine bar), which is consistent with previous studies that have reported vesicular TGF-β1 secretion [35]. However, for these EVs derived from breast cancer cells, the amount of measured vesicle TGF-β1 was not a significant contributor to the levels of TGF-β1 secreted as a result of EV stimulation. Contrary to EVs derived from breast cancer cells, EVs derived from both monocytic U937 cells and pancreatic cancer L3.6pL cells, EV^U937^ and EV^L3.6pL^, respectively, exhibited higher background levels of TGF-β1. Nonetheless, modest stimulations of TGF-β1 secretion were observed upon treatment of THP-1 cells with EVs derived from all cell lines excluding those stimulated with EV^U937^, since we measured high TGF-β1 background in EV^U937^ (Fig. 6, light green bar). Such an observation of high initial levels of extracellular TGF-β1 associated with EVs and subsequent reduction upon incubation with cells is consistent with previous reports that indicate cellular internalization of TGF-β1 [36]. This data indicates that not all EVs are poised to stimulate TGF-β1. In summary, it may be concluded that EVs from multiple cancer cell types that include breast cancer and pancreatic cancer, are potent stimulators of active TGF-β1 secretion in human THP-1 monocytes.

## Discussion

The involvement and importance of secreted EVs in tumor progression has become unquestionable over the past several years [1], [2], [37] and thus, here, we have utilized a straightforward and simple purification method to purify EVs from several sources in order to characterize their activities. Specifically, while EMMPRIN has previously been detected in EVs [38]–[40], our broad based analysis of EMMPRIN secretion further establishes the presence of extracellular full length EMMPRIN in biological fluids and on secreted EVs from multiple cell types. Therefore, our work here suggests that full length EMMPRIN serves as a general marker for the presence of secreted EVs. EVs purified here were found to be internalized into target cells, consistent with previous studies that have shown that EVs are involved in cell-cell communication and the transfer of pro-oncogenic factors [12]–[14], [41]. We also probed the stimulatory activity of purified EVs and we report an important distinction with regard to vesicle activity. Namely, our data shows a differential level of EV activity depending on the recipient cell type with monocytic cells eliciting a stronger response compared to epithelial cells. Furthermore, we found that EVs stimulate secretion of EMMPRIN itself, MMP-9, IL-6 and TGF-β1.

### Size Exclusion Chromatography as a Method to Purify Secreted EVs

Several methods are commonly employed for purification of EVs and include ultracentrifugation, sucrose density gradient fractionation as well as commercially available kits such as Exoquick [42]–[44]. We chose to purify EVs using size exclusion chromatography initially using EMMPRIN as a potential marker but further validated our methods using assays that directly characterized these EVs. These biophysical methods included NanoParticle tracking analysis, electron microscopy, fluorescence microscopy and mass spectrometry analysis. We find that size exclusion chromatography offers several advantages. For example, ultracentrifugation methods require very high speeds, i.e. 100,000×g, for several hours and likely result in the pelleting of high molecular weight soluble proteins secreted by the cells but not necessarily incorporated within the EVs. Likewise, sucrose gradient fractionation does yield pure EV fractions, however, this method also requires very long centrifugation times. Thus, size exclusion chromatography may offer a simple and potentially more advantageous method of EV purification that avoids long high-speed centrifugation steps. Subsequent mass spectrometry analysis yielded identification of markers for both exosomes and microvesicles and, therefore validated our method of EV purification. Our fluorescence microscopy data showing that vesicles are internalized within minutes into the recipient cells is also consistent with previous reports on vesicle internalization and serves as yet another method to validate our purification method [12]–[14], [41]. Thus, the subsequent activity studies we performed are a direct measure of purified EVs.

### EVs Stimulate Secretion of Molecules Upregulated in Human Cancers in a Cell-specific Manner

Our data showed that EVs purified from multiple cancer cells preferentially target monocytic cells resulting in stimulation of several factors consistently shown to be upregulated and play a role in several different cancers (Fig. S2 and Fig. S3). This finding is particularly important considering the mobility of these cells and their localization within the bloodstream [45]. Elaborating, monocytes that we have shown here to be primarily responsive to EVs are also cells of the innate immune system that can differentiate to macrophages and dendritic cells [46], [47]. Thus, our findings are consistent with the proposed phenotypic changes of these cells regulated by EVs, which alter the immune response and, therefore, the body’s ability to fight cancer [34].

The upregulated cancer inducing factors probed here include EMMPRIN, MMP-9, IL-6, and TGF-β1 and all were secreted upon stimulation with EVs used here (Fig. 3–6). We showed that while EMMPRIN may serve as a general marker for secreted EVs (Fig. 1B), we also found that these EVs stimulate the secretion of full length EMMPRIN, which we have shown here to be a marker for EVs, from the cells that they target and, thus, likely comprise a feedback loop for EVs stimulating their own expression as well (Fig. 3). MMP-9 upregulation is significant because of the role of this particular MMP in the breakdown of the extracellular matrix [18]. The breakdown of barriers preventing cancer cells from invading into secondary sites allows for progression of cancer and is mediated by the MMP family of proteins [48]. The observed increased secretion of EMMPRIN with the concomitant increase of MMP-9 secretion may imply a higher metastatic potential, since increased expression of these two proteins is correlated in several different cancers and is a poor prognostic indicator for patients [29], [31], [49]. Furthermore, the role of MMPs in development of metastasis is well established and the levels of MMP-9 in serum of cancer patients correlate with metastatic potential [50]–[52]. IL-6 is a known pro-inflammatory cytokine with an established role in tumor progression and metastasis [19], [53]. Our data is consistent with a previous report concluding that monocytes purified from patients’ peripheral blood secrete increased levels of IL-6 upon stimulation with tumor-derived EVs [35]. This may point to an important biological function of EVs in modulating a pro-inflammatory phenotype of target cells as well as establishing a cancer-promoting environment. TGF-β1, along with secreted EVs, prevents the differentiation of monocytes to dendritic cells, thereby down-regulating the immune response [35]. Thus, our observed secretion of TGF-β1 upon stimulation with EVs is once again consistent with a potential phenotype change of the THP-1 monocytic cells. In fact, such a phenotype change regulated by EVs through TGF-β1 has recently been proposed to alter the differentiation pathway of monocytes towards a more immunosuppressive cell type known as myeloid derived suppressor cells [34]. Identification of the receptors and the pathways that are involved in the EV-mediated stimulation of molecules studied here was not included in the scope of our study. However, given the vast array of EV cargo it is likely that a number of different receptors and pathways are mediating the observed stimulatory response. Identifying these receptors and pathways is an important next step toward a better understanding of EV biology.

## Supporting Information

Figure S1 **Full length extracellular EMMPRIN is detected in biological fluids.** A) Serum filtered through 0.22 µm was IPed for EMMPRIN and deglycosylated with PNGaseF. EMMPRIN was detected using Western Blot analysis. Full length EMMPRIN was detected in serum from both healthy and leukemia patients and likely secreted via EVs. B) Mass spectrometry analysis was used to confirm EMMPRIN within the human sera sample. Several bands were used in unambiguous identification of EMMPRIN in human serum and are mapped onto the EMMPRIN crystal structure.
(TIF)

Figure S2 **MMP-9 secretion upon vesicle stimulation is cell-type dependent.** MMP-9 secretion upon stimulation with EV^MDA^ and EV^MCF-7^ is shown for several different target cell lines: monocytic cells lines U937 and Molm13, a fibroblast cell line Human Foreskin Fibroblast (HFF) and epithelial cell lines MCF-7, MCF-10A and MDA-MB-231 cells. Secretion of MMP-9 is only observed in the monocytic U937 cells while epithelial cells are not responsive to vesicle stimulation.
(TIF)

Figure S3 **IL-6 secretion upon vesicle stimulation is cell-type dependent.** IL-6 secretion upon stimulation with EV^MDA^ and EV^MCF-7^ is shown for the cell lines described in Figure 2. Secretion of IL-6 is only observed in the monocytic U937 cells upon stimulation with EV^MDA^ but not with EV^MCF-7^. Among the epithelial cell lines, only the HFF cells secreted IL-6 (others not shown) upon stimulation with both EV^MDA^ and EV^MCF-7^.
(TIF)

Figure S4 **Vesicle internalization in U937 cells.** Fluorescence microscopy images of U937 cells incubated with Texas Red stained EVs. The cell membrane was stained with EMMPRIN-FITC (green) and the nucleus was stained with DAPI (blue). U937 cells were incubated for 5 minutes with Texas Red stained EVs and then visualized. The reference bar is 10 µm.
(TIF)
