# Supplementary figures and images for: Extracellular Vesicles Secreted from Cancer Cell Lines Stimulate Secretion of MMP-9, IL-6, TGF-β1 and EMMPRIN
Source: PLoS One. 2013 Aug 1;8(8):e71225. doi: 10.1371/journal.pone.0071225 (PMC3731303; doi:10.1371/journal.pone.0071225)

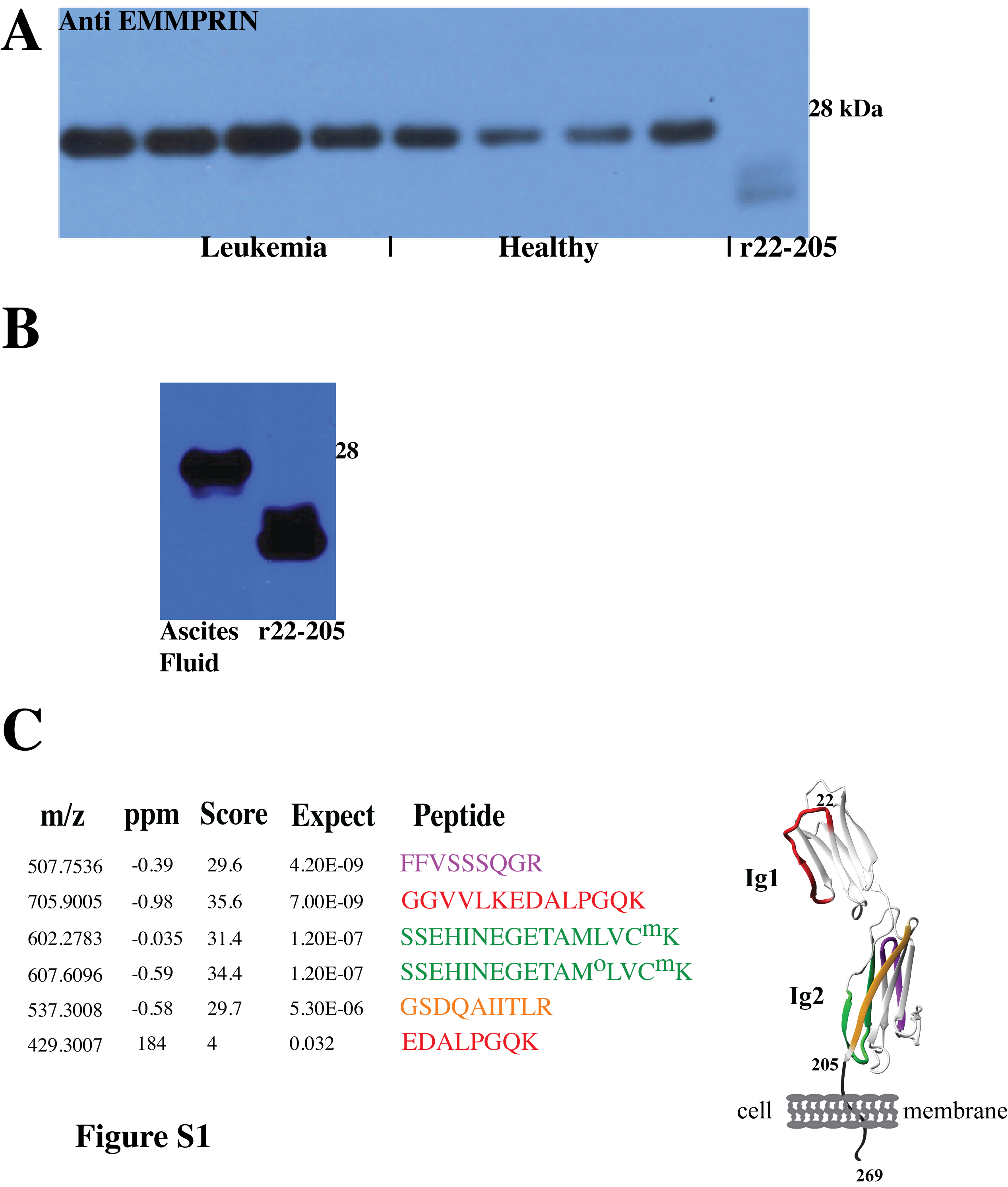

Supplement: Figure S1 — Full length extracellular EMMPRIN is detected in biological fluids. A) Serum filtered through 0.22 µm was IPed for EMMPRIN and deglycosylated with PNGaseF. EMMPRIN was detected using Western Blot analysis. Full length EMMPRIN was detected in serum from both healthy and leukemia patients and likely secreted via EVs. B) Mass spectrometry analysis was used to confirm EMMPRIN within the human sera sample. Several bands were used in unambiguous identification of EMMPRIN in human serum and are mapped onto the EMMPRIN crystal structure. (TIF) [file pone.0071225.s001.tif]

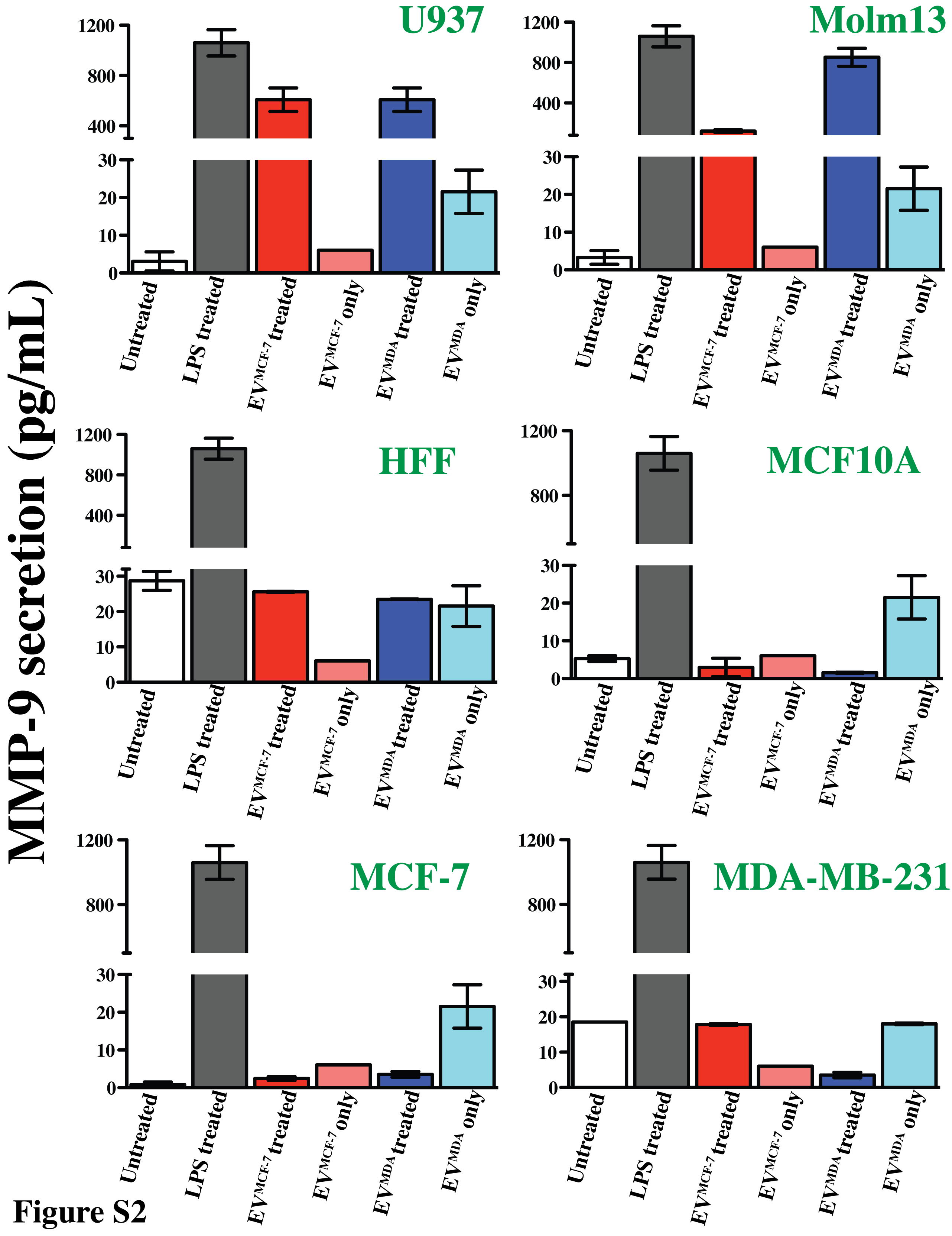

Supplement: Figure S2 — MMP-9 secretion upon vesicle stimulation is cell-type dependent. MMP-9 secretion upon stimulation with EVMDA and EVMCF-7 is shown for several different target cell lines: monocytic cells lines U937 and Molm13, a fibroblast cell line Human Foreskin Fibroblast (HFF) and epithelial cell lines MCF-7, MCF-10A and MDA-MB-231 cells. Secretion of MMP-9 is only observed in the monocytic U937 cells while epithelial cells are not responsive to vesicle stimulation. (TIF) [file pone.0071225.s002.tif]

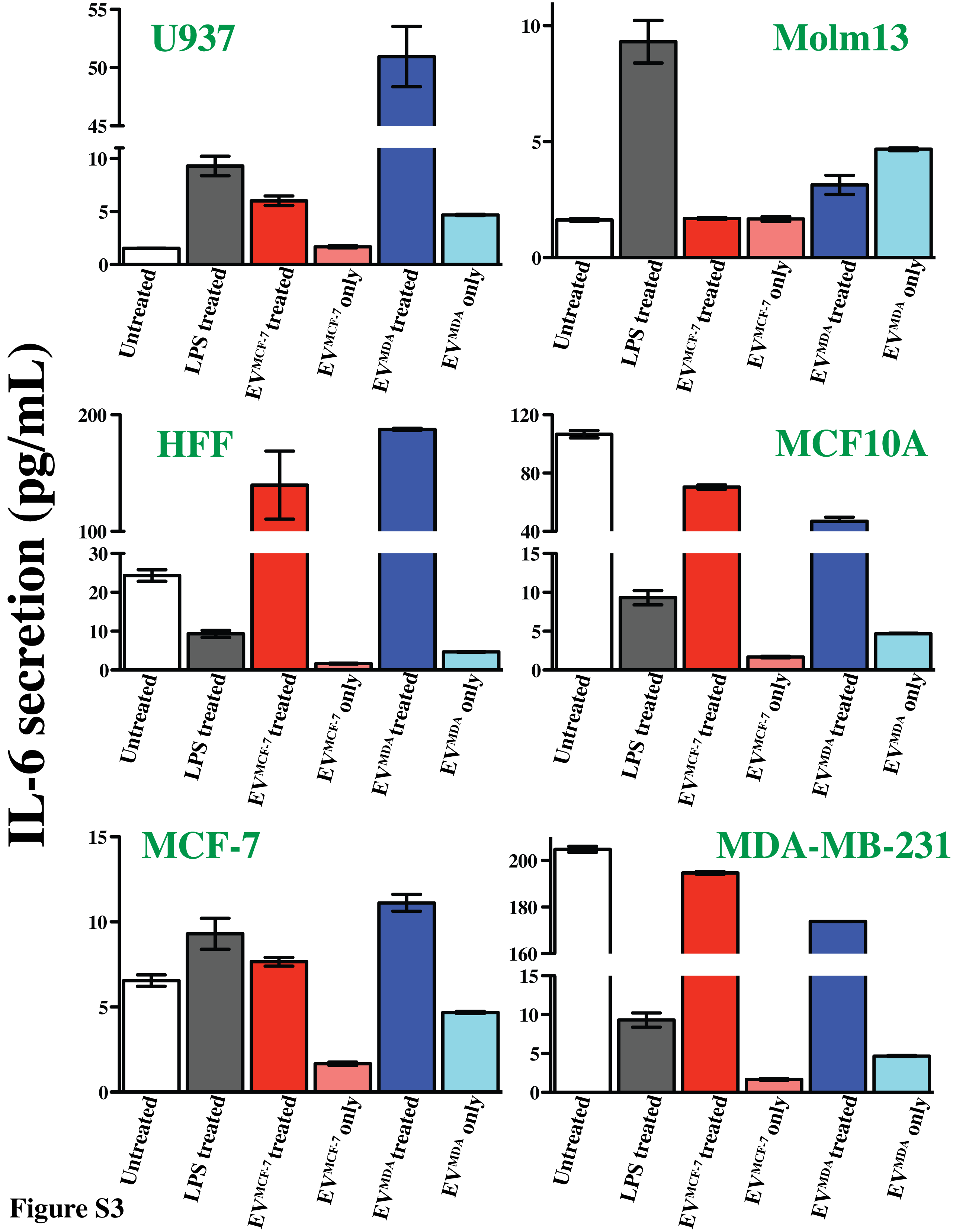

Supplement: Figure S3 — IL-6 secretion upon vesicle stimulation is cell-type dependent. IL-6 secretion upon stimulation with EVMDA and EVMCF-7 is shown for the cell lines described in Figure 2. Secretion of IL-6 is only observed in the monocytic U937 cells upon stimulation with EVMDA but not with EVMCF-7. Among the epithelial cell lines, only the HFF cells secreted IL-6 (others not shown) upon stimulation with both EVMDA and EVMCF-7. (TIF) [file pone.0071225.s003.tif]

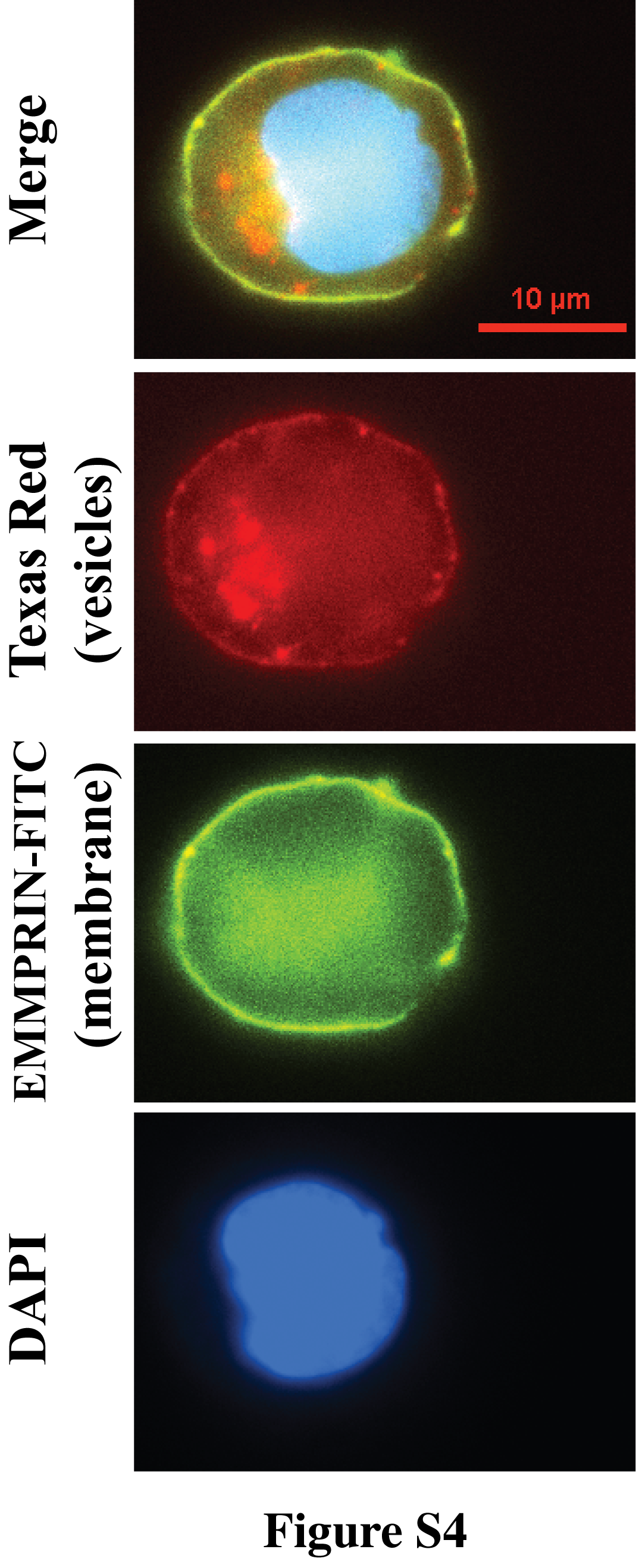

Supplement: Figure S4 — Vesicle internalization in U937 cells. Fluorescence microscopy images of U937 cells incubated with Texas Red stained EVs. The cell membrane was stained with EMMPRIN-FITC (green) and the nucleus was stained with DAPI (blue). U937 cells were incubated for 5 minutes with Texas Red stained EVs and then visualized. The reference bar is 10 µm. (TIF) [file pone.0071225.s004.tif]
